# Adenosine metabolism as an endogenous protective mechanism in response to upstream ischemic injury

**DOI:** 10.3389/fmolb.2026.1807841

**Published:** 2026-06-16

**Authors:** Liguo Zhang, Hengzhu Zhang, Shaoyan Zhou, Shuangxi Feng, Sijia Luo, Dai Wei

**Affiliations:** 1 Department of Neurosurgery, Shibei Hospital of Jing’an District, Shanghai, China; 2 The Yangzhou School of Clinical Medicine of Dalian Medical University, Yangzhou, China; 3 Northern Jiangsu People’s Hospital, Yangzhou, China; 4 Department of Equipment, Shibei Hospital of Jing’an District, Shanghai, China; 5 Procurement Center, Zhabei Central Hospital of Jing’an District, Shanghai, China; 6 Asset Management Department, Putuo District People’s Hospital, Shanghai, China

**Keywords:** adenosine metabolism, HMBS/POR, ischemic stroke, neuroprotection, oxidative stress

## Abstract

**Background:**

Following ischemic stroke (IS), endogenous protective mechanisms are activated to mitigate brain injury. Adenosine (ADO), a key endogenous immunomodulator, is implicated in this response, yet the precise regulatory pathways linking upstream ischemic injury to ADO-mediated protection remain incompletely elucidated.

**Methods:**

We integrated single-cell RNA sequencing (scRNA-seq) data (GSE174574) and bulk RNA-seq data (GSE140275) from IS models. After preprocessing and t-SNE-based clustering, we conducted analyses of cell communication and differential expression. Differentially expressed genes were intersected with ADO metabolism-related genes from the GeneCards database to identify candidate regulators. The roles of these candidates in modulating ADO metabolism, oxidative stress, and apoptosis were functionally validated *in vitro* using qRT-PCR, Western blot, ELISA, and gene silencing in BV2 cells.

**Results:**

Unsupervised clustering of scRNA-seq data identified 14 cell types, with microglia displaying extensive intercellular communication, particularly via the CCL and TNF signaling pathways. Cross-database analysis identified four candidate molecules: HMBS, UCP2, POR, and TNF. These were positively correlated with pro-ADO metabolic genes in middle cerebral artery occlusion (MCAO) models and were upregulated in LPS-stimulated BV2 cells, coinciding with increased inflammation, oxidative stress, and ADO levels. Silencing HMBS or POR reversed these effects. Exogenous ADO reduced oxidative stress and apoptosis in BV2 cells and promoted M2 microglia polarization.

**Conclusion:**

Our findings suggest that ischemic injury upregulates specific molecules (HMBS, UCP2, POR, and TNF) potentially associated with ADO metabolism, which may, in turn, alleviate oxidative stress and apoptosis while favoring anti-inflammatory microglial polarization. This supports the hypothesis that enhanced adenosine metabolism represents a potential endogenous protective mechanism activated in response to upstream ischemic insult. HMBS or POR silencing upregulated adenosine deaminase/adenosine kinase (ADA/ADK) and downregulated SLC29A1/A2 in LPS-stimulated BV2 cells. The protective effects of ADO were abolished by A_2_aR antagonist SCH 58261, confirming the functional link between HMBS/POR and ADO-mediated neuroprotection.

## Introduction

Ischemic stroke (IS) is one of the most disabling and lethal vascular diseases worldwide (Zhao et al., 2022). It is characterized by acute occlusion of cerebral blood vessels leading to localized interruption of blood flow to brain tissue, triggering a complex cascade of pathological responses (Ospel et al., 2024). In the ischemic core and semi-dark zones, a collapse in energy metabolism triggers excitatory amino acid release, Ca^2+^ overload, mitochondrial dysfunction, bursts of reactive oxygen species (ROS) production, and activation of neuroinflammation, ultimately leading to neuronal death and disruption of the blood–brain barrier (Ni et al., 2022; Li et al., 2024; Lu and Wen, 2024). Although revascularization therapy, including intravenous and endovascular thrombolysis, can partially restore blood flow, its narrow therapeutic time window and the secondary damage of oxidative stress and inflammation after reperfusion greatly limit the clinical therapeutic effect (Ma et al., 2025). Therefore, in-depth analysis of the regulatory network of endogenous neuroprotective mechanisms after cerebral ischemia is urgently needed to develop novel adjuvant therapeutic strategies.

Damaged or dead cells in IS release adenosine triphosphate (ATP), which acts as a damage-associated molecular pattern (DAMP) to activate microglia via P2 receptors, which, in turn, recruit other immune cells, culminating in an inflammatory cascade response (Kim et al., 2020). Ectonucleotidases, including CD39 and CD73, counterbalance the inflammatory response by catabolizing ATP into adenosine (ADO) (Schädlich et al., 2023). ADO has been shown to have anti-inflammatory effects, is an important endogenous protective mediator in the central nervous system (Wang et al., 2023), and changes dynamically during the pathological process of IS. Mechanistically, extracellular ADO levels are significantly elevated after IS (Boison and Shen, 2010), exerting multiple protective effects by binding to ADO A1 receptors (A1Rs), ADO A2A receptors (A_2_ARs), ADO A2B receptors (A2BRs), and ADO A3 receptors (A3Rs), including attenuating neuronal and glial damage and inhibiting neuroinflammation (Dettori et al., 2025; Liston et al., 2022).

Notably, the ADO metabolism is precisely regulated by key molecules such as ADO deaminase (ADA), ADO kinase (ADK), and nucleoside transporters (SLC29A1/A2), which are involved in the processes of ADO synthesis, ADO catabolism, and intracellular transport of ADO (Shen et al., 2011; Ling et al., 2022; Chiang et al., 2023; Zhang et al., 2020). However, the cell-specific regulatory mechanisms of ADO metabolism in the IS microenvironment, particularly the central role of MG in this network, have not yet been elucidated. MG, as resident immune cells in the brain, are rapidly activated after ischemia and undergo a dynamic shift from pro-inflammatory M1-type to anti-inflammatory M2-type. Their secretion of signaling molecules, such as CCL/TNF, may affect ADO metabolism through paracrine or autocrine pathways (Akimoto et al., 2013; Harms et al., 2012), constituting a potential endogenous neuroprotective axis.

By integrating scRNA-seq data (GSE174574) and bulk RNA-seq data (GSE140275) from public databases, the present study aimed to systematically resolve the ADO metabolism in IS cell-specific regulatory mechanisms. We revealed the centrality of MG as a hub of cellular interactions and screened key regulatory molecules, including HMBS, UCP2, cytochrome P450 reductase (POR), and TNF, using differentially expressed genes in conjunction with a set of genes related to ADO metabolism. The study further suggests the innovative hypothesis that the upregulation of HMBS, POR, and TNF directly or indirectly increases inflammation and oxidative stress on the one hand, and, on the other hand, decreases the expression of ADA and ADK and inhibits ADO catabolism. At the same time, activation of 5′, 3′-nucleotidase, cytosolic (NT5C), and SLC29A1/A2 promotes ADO synthesis and transport, ultimately elevating ADO levels to initiate the endogenous protective program. The elucidation of this injury-response-protection mechanism not only deepens the understanding of the pathophysiology of IS but also provides a theoretical basis for developing neuroprotective agents targeting the ADO metabolic pathway and candidate targets.

## Methods

### Data sources and preprocessing

The scRNA-seq data (GSE174574) used in this study were obtained from the Gene Expression Omnibus (GEO) database. Data were sorted according to sample number, and all analyses were carried out in the R (version 4.4.1) and Seurat (version 5.1.0) environments. Each sample was read with the Read10X function, and a separate Seurat object was generated using the CreateSeuratObject function, with the filtering criteria set to at least 300 genes detected per cell and genes expressed in at least five cells. Afterward, the merge function is used to integrate all the sample data and generate a unified Seurat object for subsequent analysis.

### Quality control and data filtering

As part of the quality control pipeline, we calculated and assessed the percentage of reads originating from mitochondrial, ribosomal, and hemoglobin genes. The percentage in each cell was calculated separately (PercentageFeatureSet achieved), and the filtering criteria were that mitochondrial genes accounted for <20% for mitochondrial genes, >3% for ribosomal genes, and <1% for hemoglobin genes. The feature genes were required to be expressed in at least three cells, and the number of genes detected in each cell was less than 4000. High-quality cells retained after the above quality control and filtration were used for subsequent analyses.

### Data normalization, batch effect correction, and dimensionality reduction

The filtered data were LogNormalize normalized using the NormalizeData function (scale.factor = 1e4), and highly variable genes were screened using FindVariableFeatures, followed by ScaleData normalization. To eliminate the batch effect among different samples, Harmony correction was performed using the RunHarmony function, and nonlinear downscaling and visualization were performed by RunUMAP and RunTSNE, respectively.

### Cluster analysis and cell-type annotation

Cell clustering was performed using the FindClusters function in the Seurat package with resolution = 0.2 and the Louvain algorithm for community detection. Dimensionality reduction was performed using the top 20 principal components (PCs) determined by the elbow plot method. Biological annotation of the cell clusters was performed in combination with published literature and canonical cell-type marker genes to identify cells including astrocytes (ASCs), choroid plexus capillary endothelial cells (CPCs), endothelial cells (ECs), ependymocytes (EPCs), fibroblasts (Fibr), lymphocytes (Lym), macrophages (MACs), monocyte-derived cells (MdCs), MG, neutrophils (NEUT), neural progenitor cells (NPCs), oligodendrocytes (OLGs), pericytes (PCs), and vascular smooth muscle cells (SMCs). Annotation information was written to meta.data and used for subsequent visualization and downstream analysis.

### Dimensionality reduction and visualization

We performed principal component analysis (PCA) with the RunPCA function and identified the optimal number of principal components by examining the elbow plot. Based on the Harmony correction results, RunUMAP and RunTSNE were used for nonlinear dimensionality reduction and visualization, and the accuracy of cell classification was verified by a DimPlot, a violin plot, and a gene expression scatter plot.

### Cell communication analysis and microglia differential analysis

To systematically analyze the dynamics of the intercellular communication network in the middle cerebral artery occlusion (MCAO) model, single-cell data from the MCAO and Sham groups were extracted based on the Seurat object. The ligand-receptor interaction network was inferentially analyzed using the CellChat (version 2.1.2) R package, and the database was selected as CellChatDB.mouse and restricted to the “Secreted Signaling” pathway. Data preprocessing included gene overexpression and interaction pair identification. Intercellular communication probabilities were calculated by the computeCommunProb and computeCommunProbPathway functions, and the aggregateNet summary network was used. The netVisual_circle, netVisual_ heatmap, and netAnalysis_contribution functions were used for communication network visualization.

To further reveal the transcriptional signature changes of MG in cerebral ischemic injury, MG cell subpopulations were extracted based on the annotation information. Differential expression analysis of microglia (MG) between the MCAO and Sham groups was performed using the FindMarkers function. The screening thresholds for significant DEGs were set as: avg_log2FC ≥ 0.25 and min.pct ≥ 0.25, with p-values calculated using the Wilcoxon rank-sum test. Significant genes were used for subsequent functional validation and visualization.

### Differential expression analysis and correlation analysis of bulk RNA-seq

The original reads count matrix was first quality controlled using R (version 4.4.1) to filter low-expression genes with read sums less than 10 across all samples. The duplicate genes were de-weighted according to the total counts of each gene, and the transcript with the highest expression was retained as the representative gene. Differential expression analysis was performed using DESeq2 (version 1.44.0). A DESeqDataSet object was constructed based on sample grouping information, and read counts were normalized by geometric mean normalization. Significance was determined using the Wald test for the negative binomial distribution, followed by Benjamini–Hochberg (BH) correction for multiple testing. Significant DEGs were defined as |log_2_FoldChange| ≥ 1 and adjusted p-value (padj) < 0.05.

Pearson correlation coefficients were calculated using bulk RNA-seq data. Scatter plots with regression lines and 95% confidence intervals were generated.

### Multi-omics integration and screening of genes related to ADO metabolism

To further explore the key genes that may be involved in the regulation of ADO metabolism in cerebral ischemia from a multidimensional perspective, the present study combined bulk RNA-seq, microglia scRNA-seq, and GeneCards database “ADO metabolism” related gene sets to perform intersection analysis. The bulk RNA-seq differential gene screening threshold was log_2_FoldChange ≥ 1 and padj <0.05, and the MG cell subpopulation differential gene screening threshold was avg_log2FC ≥ 1 and p-value <0.05. We searched the GeneCards platform with the search keyword “ADO metabolism” in the GeneCards platform and extracted metabolism-related genes with a relevance score ≥ 15. The intersection operation was performed sequentially to obtain a high-confidence candidate gene set. The intersected genes were descending-ordered by log_2_FoldChange and avg_log2FC to highlight the key genes. Finally, the Venn diagram was drawn using the VennDiagram package to show the overlapping relationship of genes.

### Cell culture and transfection

Mouse microglial cell lines (BV2 cells) were cultured in DMEM supplemented with 10% fetal bovine serum (FBS) and 1% penicillin–streptomycin at 37 °C in an atmosphere of 5% CO_2_: sh-HMBS#1 5′-GGAUCAAGUUCUCCGAGUAU-3′ and sh-HMBS#2 5′-AUACUCGGAGAACUGAUCCU-3′, sh-POR#1 5′-GCACAGCUGUGAAGUCAAUU-3′ and sh-POR#2 5′-UUGACUUCACAAGCUGUCCUUU-3′. The shRNA negative control (sh-NC, sequence: ATCG GTAGCTCCATTACGCGT) in the 1-puro vector was obtained from HonorGene (Beijing, China). Cell transfection was carried out using Lipofectamine 2000 (Invitrogen, Shanghai; Cat #11668030) according to the manufacturer’s protocol. Following stable integration, cells were selected with 1 μg/mL puromycin for 14 days prior to subsequent experiments. BV2 cells were purchased from the Cell Bank of the Chinese Academy of Sciences and authenticated by STR profiling. The LPS treatment strategy (500 ng/mL, 3 h) was selected based on widely validated protocols for microglial inflammatory activation in ischemic stroke studies (Jia et al., 2023; Yuan et al., 2016; Martins et al., 2021), which stably induce oxidative stress and inflammatory responses without overt cytotoxicity.

For exogenous ADO treatment, LPS-stimulated BV2 cells were incubated with 100 μM ADO for 24 h. This concentration was selected based on widely validated protocols in microglia studies (Lin et al., 2008; Cheng X. et al., 2022): 100 μM ADO consistently exerts anti-inflammatory, antioxidant, and anti-apoptotic effects without inducing cytotoxicity. For A_2_aR inhibition, BV2 cells were pretreated with SCH 58261 (50 nM) for 1 h before LPS and ADO treatment, as previously described (Cheng Y. et al., 2022).

### Enzyme-linked immunosorbent assay

BV2 cells were inoculated into 12-well plates at 5 × 10^5^ cells per well. After different treatments, cell cultures of each group of cells were collected and centrifuged to obtain supernatants. The levels of several secreted proteins, including TNF-α (JL10484), IL-6 (JL20268), IL-1β (JL18442), IL-10 (JL20242), and Arg-1 (JL13668), were detected in the supernatants according to the methods provided in the manual. The ELISA kits were purchased from JONLNBIO.

### Quantitative real-time PCR (qRT-PCR)

BV2 cells were inoculated into 6-well plates at 1 × 10^6^ cells per well. After different treatments, the cells were harvested for extracting total RNA, and the RNA concentration was determined using a NanoPhotometer. The cDNA (Vazyme, R323-01) was synthesized using 1 μg of total RNA according to the manufacturer’s instructions, followed by a 10-fold dilution in ddH2O. Then, qRT-PCR (Vazyme, Q321-02) was performed. HMBS forward primer: CTGGTGATGGTGTTGGAGGA; HMBS reverse primer: CCAGGAAGCGGGTAGAGTTC. UCP2 forward primer: CTTCCACCTCCAGCTCACTG; UCP2 reverse primer: GGTAGGCTGCCAGATCTTCC. POR forward primer: TGGCTGTTGTTGGGATTCTG; POR reverse primer: TCCACAGCCACCATCTTCAC. TNF-α forward primer: CCCTCACACTCAGATCATCTTCT; TNF-α reverse primer: GCTACGACGTGGGCTACAG. GAPDH forward primer: GCAAATTCCATGGCACCGT; GAPDH reverse primer: TCGCCCCACTTGATTTTGG. ADA forward primer: GGAGCAGGCTCATCGAGAAG; ADA reverse primer: ACCGCACCTCCACATACACC. ADK forward primer: CCTCTATGAGTTGAGATCCTGTCTCC; ADK reverse primer: ATTTATTAACTTTACATAGATTCAGACAG. NT5C forward primer: GCGGTACTTGGGATAATAGA; NT5C reverse primer: ATAAAGATGAATCGGCATTG. SLC29A1 forward primer: CTTGGGATTCAGGGTCAGAA; SLC29A1 reverse primer. ATCAGGTCACACGACACCAA; SLC29A2 forward primer: CATGGAAACTGAGGGGAAGA; SLC29A2 reverse primer: GTTCCAAAGGCCTCACAGAG.

### The detection of cellular ROS

BV2 cells were inoculated into 12-well plates at 5 × 10^5^ cells per well. After different treatments, the culture medium was removed and washed three times with PBS. Then, 500 μL of 2′,7′-dichlorodihydrofluorescein diacetate (DCFH-DA) working solution (Servicebio, G1706) was incubated with BV2 cells for 30 min in a cell culture incubator protected from light. The working solution was removed after 30 min, and the sample was washed three times with PBS. Finally, the mean fluorescence intensity (MFI) was quantified using ImageJ.

### The detection of cell apoptosis

We seeded BV2 cells in 12-well plates at 5 × 10^5^ cells per well. After treatment, the medium was discarded, and the cells were subjected to three washes with PBS. Cells were fixed for 30 min using 4% paraformaldehyde (PFA) and permeabilized for 5 min using 0.3% Triton X-100 in sequence according to the method provided in the instruction manual (Beyotime, C1089). The prepared TUNEL assay solution was then incubated with the cells for 60 min at 37 °C, protected from light. After 30 min, the cells were washed three times with PBS. Finally, the cells were incubated with an anti-fluorescence quenching sealer containing DAPI (Servicebio, G1407) for 10 min, and images were obtained using fluorescence microscopy.

### Statistical analysis

All *in vitro* experiments were performed with at least three independent biological replicates (n ≥ 3). Data are expressed as the mean ± SD (M ± SD). Differences between groups were assessed with Student’s t-test or one-way ANOVA (with Tukey’s *post hoc* test for multiple comparisons) in GraphPad Prism 10.1.2. Statistical significance was set at *p < 0.05, **p < 0.01, ***p < 0.001, and ****p < 0.0001.

## Results

### Quality control and sample integration of scRNA-seq data

**FIGURE 1:**
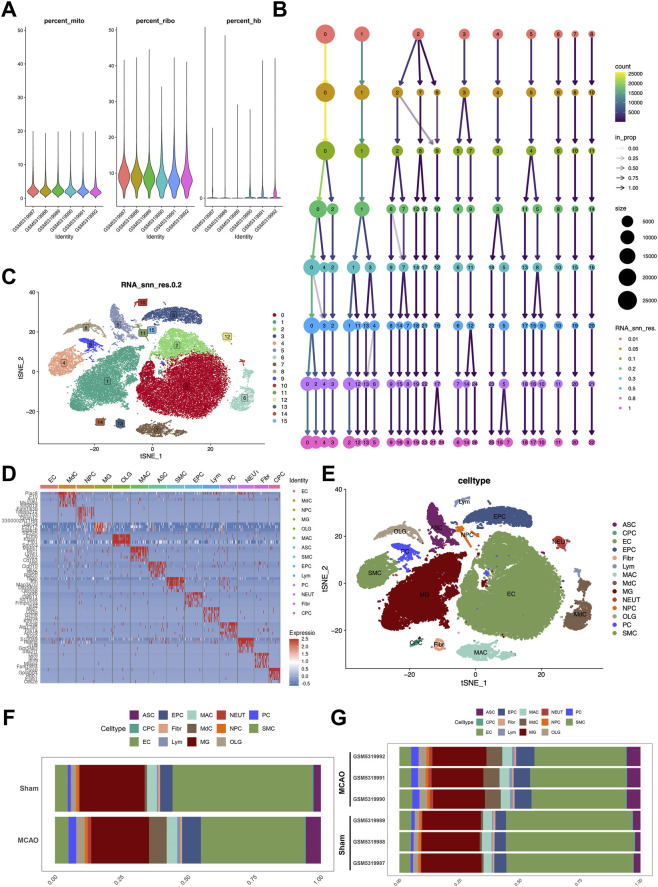
QC, integration, clustering, and cell-type annotation of scRNA-seq data. **(A)** Violin plots showing the distribution of mitochondrial (percent_mito), ribosomal (percent_rbo), and hemoglobin (percent_hb) gene expression ratios across samples, serving as core quality control metrics to validate scRNA-seq data integrity and reliability. **(B)** Hierarchical clustering tree of cells at varying clustering resolutions after Harmony-based batch integration, used to select the optimal resolution balancing clustering granularity and biological rationality. **(C)** t-SNE visualization of integrated scRNA-seq data, identifying 16 unsupervised cell clusters (0–15) with distinct colors, illustrating cell population distribution and inter-cluster transcriptomic similarity for subsequent cell-type annotation. **(D)** Heatmap of the top five specific marker genes for each cluster, with color gradients indicating normalized gene expression (red: high, blue: low), enabling cell identity assignment via canonical marker expression patterns. **(E)** t-SNE visualization of cell-type annotation, with colors representing major cell types (e.g., ASCs, ECs, and CPCs), mapping unsupervised clusters to biologically meaningful cell subpopulations. **(F)** Stacked bar plot of cell population proportions at the tissue level, showing the abundance distribution of each cell subpopulation. **(G)** Stacked bar plot of cell population proportions across samples, revealing disease-induced changes in tissue cell composition and providing a basis for downstream functional analysis of differential cell subpopulations.

First, we performed stringent quality control on the scRNA-seq data (Figure 1A). The number of genes detected per cell (nFeature_RNA) should be less than 4000; the percentage of mitochondrial gene expression (percent_mito) should be less than 20%; the percentage of ribosomal protein gene expression (percent_ribo) should be (percent_mito) less than 20%, percent_ribo (percent_ribosomal_protein) should be less than 3%, and percent_hb (percent_hb) should be less than 1%. The remaining cells were used for subsequent analyses.

Sixteen clusters (Figure 1C) and their markers (Figure 1D) were initially obtained by combining different samples after correcting for batch effects using the Harmony algorithm (Figure 1B) and after dimensionality reduction using the t-SNE algorithm and unsupervised clustering. Then, we further manually annotated 14 cellular subgroups (Figure 1E), including ASCs, CPCs, ECs, EPCs, Fibr, Lym, MACs, MdCs, MG, NEUT, NPCs, OLGs, PCs, and SMCs, and identified them in a sham-operated (Sham) group and a MCAO group (Figure 1F) and their relative proportion in individual samples (Figure 1G). Among them, ECs showed the highest relative proportion, followed by MG. The relative proportion of EC in the MCAO group was lower than that in the Sham group, suggesting that IS induced intense vascular endothelial injury. In contrast, the relative proportion of MG in the MCAO group was lower than that in the Sham group, which was probably caused by a decrease in the number of resident microglial cells resulting from a large immune cell infiltration.

### MG is the core cellular subpopulation in IS

**FIGURE 2:**
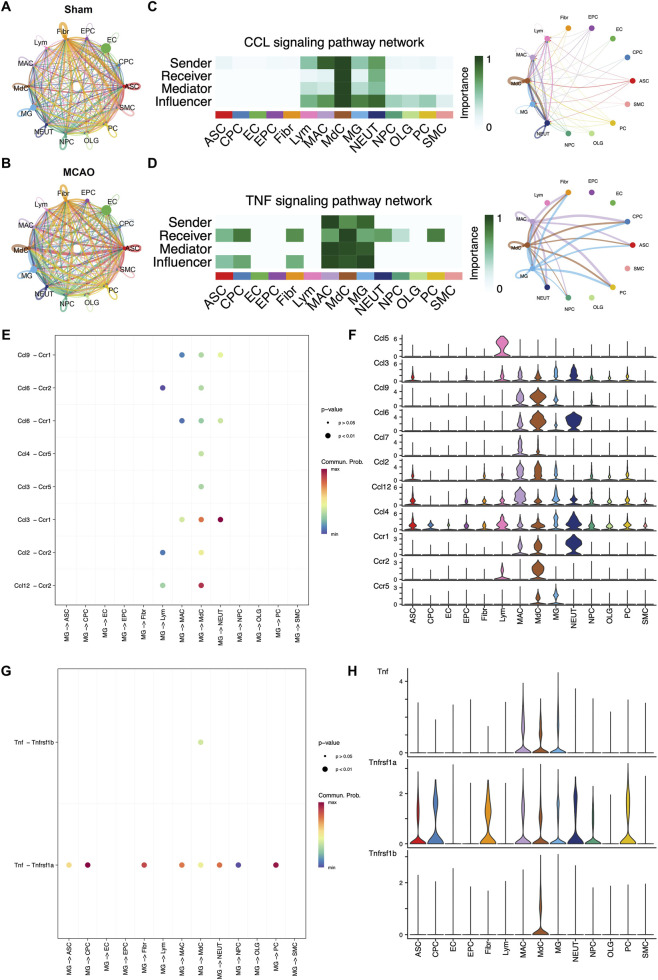
MG is the core cell subpopulation in IS. Illustration of signal crosstalk via the circle diagram in the **(A)** Sham group and **(B)** MCAO group. Thickness represents signal intensity. **(C)** Heatmap and network of senders, receivers, regulators, and influencers of the CCL signaling pathway. **(D)** Heatmaps and networks of senders, receivers, regulators, and influencers of the TNF signaling pathway. **(E)** Significance bubble diagram of CCL ligand–receptor pairs and their communication probabilities. **(F)** Violin plots of the expression of key ligand and receptor genes in the CCL signaling pathway in different cell types. **(G)** Significance bubble plot of ligand–receptor pairs of the TNF signaling pathway and their communication. **(H)** Violin plots of the expression of TNF signaling pathway-related genes in various cell types.

To further investigate the role of MG in IS, we analyzed the interactions of the MG population with 13 other cell groups. Compared with the Sham group, the number of cell–cell interactions and signal intensity in the MCAO group changed significantly (Figures 2A,B). Notably, the MG population is one of the major communication hubs in the IS. In addition, the MG population also showed significant interactions with other cell populations in the CCL signaling pathway network and the TNF signaling pathway network (Figures 2C,D). Intensive exploration of CCL and TNF signaling suggests that ligand-receptor interactions from MG to other cell types contribute to signaling. For CCL signaling, notable interactions are Ccl3/Ccl6/Ccl9-Ccr1, Ccl6/Ccl2/Ccl12-Ccr2, and Ccl3/Ccl4-Ccr5 (Figures 2E,F). For TNF signaling, the interaction of Tnf–Tnfrsf1a was the most significant (Figures 2G,H). These results re-emphasize the important role of MG in the pathophysiological process of IS.

The LPS-induced BV2 inflammation model simulates the inflammatory activation and oxidative stress state of microglia after ischemic injury, which is consistent with the CCL/TNF signaling-dominated microglial communication network identified by scRNA-seq. Silencing HMBS or POR significantly reduced TNF expression in LPS-stimulated BV2 cells (Supplementary Figure S1C), directly linking the scRNA-seq-predicted TNF signaling to *in vitro* functional validation.

### The identification of the core regulator of ADO metabolism in IS

**FIGURE 3:**
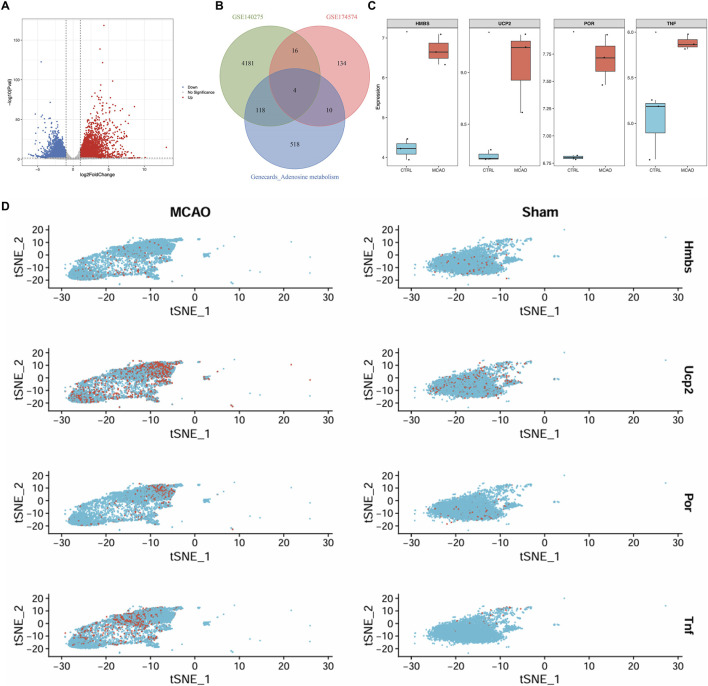
Identification of the core regulator of ADO metabolism in IS. **(A)** Volcano plot of DEGs in the bulk RNA-seq data. **(B)** The Venn diagram shows the overlapping genes of the DEGs from scRNA-seq data and bulk RNA-seq data and the gene set related to ADO metabolism from the GeneCards database. **(C)** The mRNA expression levels of HMBS, UCP2, POR, and TNF in the MCAO and Sham groups. **(D)** The t-SNE plots of HMBS, UCP2, POR, and TNF in the MCAO and Sham groups.

Bulk RNA-seq similarly revealed extensive gene expression differences between the Sham group and the MACO group. The volcano plot (Figure 3A) displays 8953 differentially expressed genes (DEGs, |log2FoldChange| ≥ 1 and p-value<0.05), with 4334 upregulated genes and 4619 downregulated genes. ADO is elevated in the tissue microenvironment in a variety of diseases and has been shown to play neuroprotective and anti-inflammatory roles in IS (Pasquini et al., 2020), which may be an endogenous protective mechanism induced by injury, including oxidative stress and inflammation. To investigate this endogenous protective mechanism and identify potential therapeutic targets for IS, we combined 4319 upregulated DEGs from bulk RNA-seq data (log2FoldChange ≥ 1 and padj<0.05) and 164 upregulated DEGs from the MG population (avg_log2FC ≥ 1 and p_val_adj<0.05) in the scRNA-seq data, combined with the gene set of genes related to ADO metabolism in the GeneCards database, which contains 650 genes. Through overlapping, we obtained four candidate molecules (Figure 3B), namely, HMBS, UCP2, POR, and TNF. The elevated expression levels of HMBS, UCP2, POR, and TNF in the MCAO group from bulk RNA-seq (Figure 3C) and scRNA-seq (Figure 3D) were demonstrated.

We prioritized HMBS and POR for functional validation because they are core upstream regulators of oxidative stress in ischemic injury: HMBS mediates heme biosynthesis and ROS accumulation, while POR is a major source of mitochondrial ROS. Both directly trigger the initial injury signal that activates ADO metabolism. In contrast, UCP2 has been proven to be a downstream compensatory protective molecule, and TNF is a pro-inflammatory effector downstream of oxidative stress. TNF and UCP2 mRNA were downregulated after HMBS silencing and POR silencing (Supplementary Figures S1A-D), suggesting a regulatory effect.

### Identified regulators may elevate the level of ADO

**FIGURE 4:**
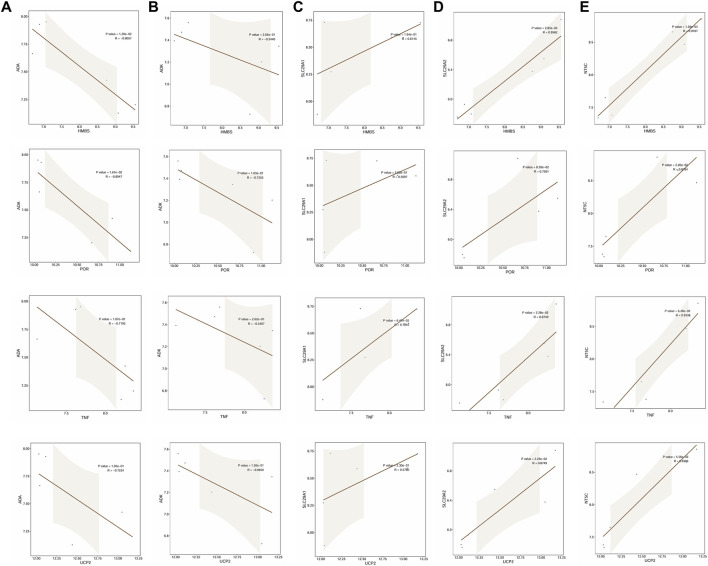
Pearson correlation analysis of batch RNA-seq. **(A)** Correlation analysis of ADA and HMBS, POR, TNF, and UCP2. **(B)** Correlation analysis of ADK and HMBS, POR, TNF, and UCP2. **(C,D)** Correlation analysis of SLC29A1/A2 and HMBS, POR, TNF, and UCP2. **(E)** Correlation analysis of NT5C and HMBS, POR, TNF, and UCP2.

To explore the potential association of the four candidate molecules we identified with elevated ADO levels in the IS tissue microenvironment and their endogenous protective mechanisms, we correlated these four molecules with ADO metabolism-related genes, including ADA, ADK, SLC29A1, SLC29A2, and NT5C, based on the bulk RNA-seq data. ADA irreversibly converts ADO to creatinine (Kutryb-Zajac et al., 2020); ADK catalyzes the metabolism of ADO to 5′monophosphate ADO (Martins et al., 2021); SLC29A1/A2 functionally promotes the intracellular transport of ADO (Best et al., 2018; Puebla et al., 2008; Morote-Garcia et al., 2009); and NT5C hydrolyzes monophosphate adenosine (AMP) to ADO (Kviklyte et al., 2017). The results showed that the four candidate molecules were negatively correlated with the expression levels of ADA (Figure 4A) and ADK (Figure 4B), suggesting reduced ADO consumption. Meanwhile, they were positively correlated with the expression levels of SLC29A1/A2 and NT5C (Figures 4C–E).

**FIGURE 5:**
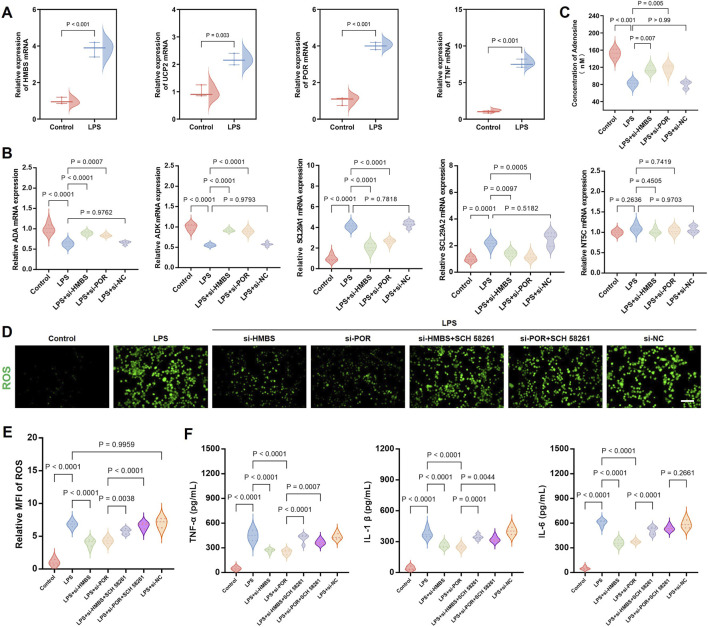
HMBS and POR-mediated oxidative stress and inflammation, triggering enhanced ADO metabolism. **(A)** The mRNA expression level of HMBS, UCP2, POR, and TNF (n = 3). LPS upregulated mRNA expression levels of HMBS, UCP2, POR, and TNF in BV2 cells. **(B)** mRNA expression level of ADA, ADK, SCL29A1, SLC29A2, and NT5C after HMBS and POR silencing in LPS-induced BV2 cells (n = 5). **(C)** HMBS and POR silencing upregulated the levels of ADO (n = 3). **(D)** HMBS and POR silencing suppressed ROS levels in LPS-induced BV2 cells, and **(E)** the relative MFI was quantified (n = 5, scale bar = 100 μm). **(F)** The level of the pro-inflammatory cytokines, including TNF-α, IL-1β, and IL-6, was detected by ELISA (n = 5). *p < 0.05, **p < 0.01, ***p < 0.001, ****p < 0.0001.

These results suggest that their high expression may promote ADO production and intracellular transport of ADO while reducing ADO consumption, thereby increasing ADO levels. To validate this correlation, we performed qRT-PCR in LPS-stimulated BV2 cells and found that silencing HMBS or POR significantly upregulated ADA and ADK expression, while downregulating SLC29A1 and SLC29A2 (Figure 5B). No significant change in NT5C expression was observed, indicating that HMBS/POR mainly regulate ADO metabolism by inhibiting catabolism and promoting transport.

More importantly, elevated ADO levels in the IS tissue microenvironment have been suggested to be an endogenous protective mechanism, whereas these four candidate molecules (HMBS, UCP2, POR, and TNF) are highly correlated with the tissue injury process. Among them, the upregulation of HMBS may lead to intracellular accumulation of hemoglobin (Moreno-Navarrete et al., 2017), which is a pro-oxidant (Prestes et al., 2020) that catalyzes lipid peroxidation and protein oxidation, thereby exacerbating the already severe oxidative stress after IS (Li et al., 2023). Similarly, POR is an important source of intracellular ROS (Reczek et al., 2017). The upregulation of HMBS and POR leads to increased oxidative stress, which, in turn, activates pro-inflammatory cytokines such as TNF and inflammatory signaling pathways. In contrast, the upregulation of UCP2 in IS may act similarly to ADO as an endogenous protective mechanism, inhibiting apoptotic processes by uncoupling the respiratory chain for reduced mitochondrial ROS production (Kretzschmar et al., 2016; Ge et al., 2017). Overall, enhanced ADO metabolism in IS may be an endogenous protective mechanism triggered by upstream increases in oxidative stress and inflammation to prevent further damage.

### The upregulation of HMBS and POR increased the level of oxidative stress and inflammation

To validate our conclusions based on bioinformatics analyses, we first constructed a model of inflamed BV2 cells *in vitro* using LPS (500 ng/mL, 3 h) (Hu et al., 2025). HMBS, UCP2, POR, and TNF-α were all upregulated in response to inflammation (Figure 5A). We then silenced HMBS and POR by LPS-treated BV2 cells to determine whether their upregulation increased oxidative stress and inflammation levels. The results showed that both oxidative stress levels (Figures 5D,E) and pro-inflammatory cytokine (TNF-α, IL-6, and IL-1β) levels (Figure 5F), which were significantly elevated by LPS treatment, were inhibited by HMBS silencing and POR silencing, respectively. Meanwhile, ADO levels were also upregulated following HMBS and POR silencing (Figure 5C). Following HMBS and POR silencing, the expression of ADO metabolism-related genes was changed (Figure 5B), and ADO levels were upregulated (Figure 5C). SCH 58261, an A_2_aR antagonist, reversed the protective effects conferred by si-HMBS and si-POR (Figure 5D-F). These results indicate that HMBS and POR may regulate oxidative stress and inflammatory responses in BV2 cells by modulating ADO metabolism.

### Exogenous ADO exerts a protective effect on LPS-treated BV2 cells

**FIGURE 6:**
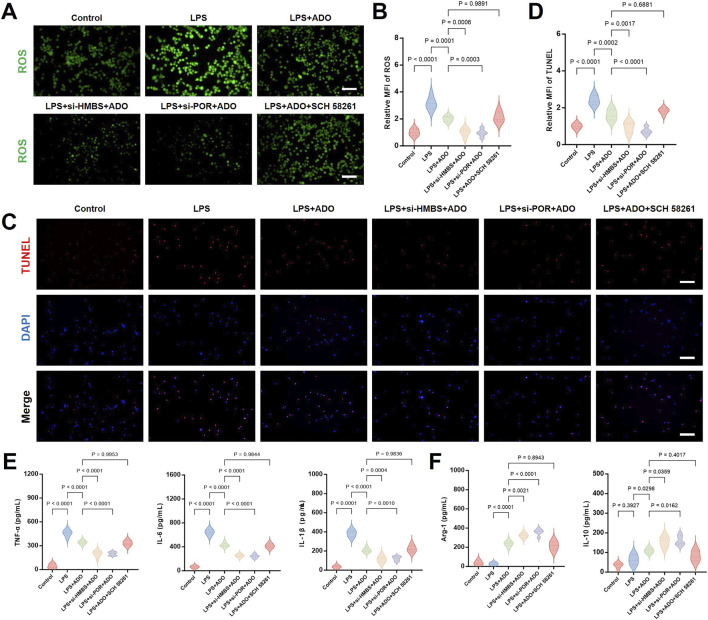
Protective effect and mechanism of ADO. **(A)** ROS level of each group and **(B)** its statistical analysis (n = 5, scale bar = 100 μm). HMBS and POR silencing further suppressed ROS levels in BV2 cells, as assessed by ADO, and SCH 58261 reversed the ADO-induced decrease in the ROS levels. **(C)** Level of cell apoptosis of each group detected using a TUNEL assay and **(D)** its statistical analysis (n = 5, scale bar = 200 μm). **(E)** Level of M1 microglia markers, including TNF-α, IL-6, and IL-1β (n = 5). **(F)** Level of M2 microglia markers, including IL-10 and Arg-1 (n = 5). *p < 0.05, **p < 0.01, ***p < 0.001, ****p < 0.0001.

Further, we treated LPS-induced inflammatory BV2 cells with exogenous ADO and found that exogenous ADO not only reduced the level of oxidative stress in inflammatory BV2 cells (Figures 6A,B) but also inhibited LPS-induced apoptosis (Figures 6C,D). In addition, exogenous ADO decreased the levels of pro-inflammatory cytokines associated with M1 microglia activation, including TNF-α, IL-1β, and IL-6 (Figure 6E). It increased the expression levels of IL-10 and Arg-1 (Figure 6F), which implied that ADO acted as an immunomodulatory agent to promote M2 polarization in M1 microglia. However, SCH 58261 reversed these effects. In addition, si-HMBS and si-POR further suppressed oxidative stress (Figures 6A-B), apoptosis (Figure 6C-D), and inflammation (Figure 6E) in response to ADO treatment, thereby enhancing the protective effects. These results further demonstrate that ADO may act as a downstream effector of HMBS and POR, regulating oxidative stress, apoptosis, and inflammatory responses in microglia.

## Discussion

In this study, we first systematically revealed the central hub role of MG in the pathological microenvironment of IS by integrating scRNA-seq data and bulk RNA-seq data. Notably, we focused our cell communication analysis on CCL and TNF signaling, the only two pathways functionally linked to microglial inflammatory activation and subsequent regulation of adenosine metabolism, rather than presenting non-specific global interactions. This targeted interpretation ensures that our single-cell data directly support the core adenosine metabolic axis of the study. MG was not only driven by extensive cell-to-cell communication through the CCL/TNF signaling network but was also identified as a key node in the metabolic regulation of ADO.

For the first time, we identified a molecular module comprising HMBS, UCP2, POR, and TNF that synergistically responds to upstream injury signals in IS, including HMBS and POR-mediated ROS bursts and TNF-dominated neuroinflammatory cascade responses. These signals increase ADO levels by negatively regulating ADO catabolic enzymes (ADA and ADK) and positively promoting ADO synthesis (NT5C) and ADO transport (SLC29A1/A2). This finding provides molecular evidence for the “injury-response-protection” axis: that is, ischemic injury signals are not purely pathogenic but instead trigger neuroprotective programs by activating endogenous ADO metabolism.

Subsequently, to validate the conclusions drawn from bioinformatics analyses through *in vitro* experiments, we found that the expression levels of HMBS, UCP2, POR, and TNF-α were elevated in LPS-treated BV2 cells, as were the levels of oxidative stress and pro-inflammatory markers. However, silencing HMBS and POR reversed the changes, demonstrating that HMBS and POR-mediated oxidative stress increased inflammation, thereby triggering increased ADO metabolism. Meanwhile, exogenous ADO significantly reduced oxidative stress and apoptosis in inflammatory BV2 cells. The decreased levels of M1 microglia markers (TNF-α, IL-6, and IL-1β) and the increased levels of M2 microglia markers (IL-10 and Arg-1) demonstrated that ADO promoted the M2 polarization of BV2 cells. These results demonstrate the protective effects and protective mechanisms of ADO.

At the mechanistic level, TNF plays a dual role in this network: as a classical pro-inflammatory factor, it exacerbates blood–brain barrier disruption and neuronal apoptosis; however, the present study demonstrates that it is also a key inducer of ADO metabolism, orchestrating neuroprotective functions of MG via autocrine/paracrine pathways. This paradoxical function is related to the spatiotemporal-specific activation of ADO receptors: elevated ADO inhibits M1 polarization of MG via A_2_AR, while attenuating excitotoxicity via A1R, forming a negative feedback regulatory loop. Notably, HMBS/POR-driven oxidative stress, although directly contributing to lipid peroxidation and mitochondrial damage, induces UCP2 expression to inhibit ROS generation, revealing a dynamic game of oxidative-antioxidant balance in the IS.

Compared with previous studies, this study clarifies that MG serves as the core of ADO metabolic regulation, remodeling the dominant immune microenvironment through ligand–receptors such as CCL3–CCR1 and TNF–TNFR1. The innovative inclusion of HMBS, UCP2, and POR into the ADO metabolic regulatory network also extends the theoretical framework of metabolic-immune crosstalk. Together, these findings suggest that both oxidative stress and inflammation may activate HMBS, POR, and TNF, which, in turn, cause ADO accumulation and trigger endogenous neuroprotective mechanisms. Targeting HMBS/POR-mediated ADO metabolism may represent a novel adjuvant neuroprotective strategy for IS, pending further *in vivo* validation.

The limitations of this study are that the role of key regulatory molecules on ADO metabolism is only based on transcriptome correlation analysis and *in vitro* experiments, and lacks *in vivo* functional experiments. The detection of ADO dynamics is limited to indirect gene correlation, and direct measurement of ADO concentration in ischemic brain regions will be required in the future. The neuroprotective mechanism of UCP2 may exist independently of the ADO pathway and must be further resolved in conditional knockdown models. scRNA-seq analysis of cellular communication failed to distinguish the M1/M2 subgroups of MG, which may mask its functional heterogeneity on ADO metabolism. The LPS-treated BV2 system is an *in vitro* single-cell model that captures microglial inflammatory activation but cannot fully recapitulate the *in vivo* microglial heterogeneity, multicellular communication, and complex tissue microenvironment inferred from scRNA-seq analyses. Thus, the conclusions are constrained within this experimental framework. Finally, the study did not explore the interventional effects of clinical reperfusion therapies, such as thrombolysis or thrombectomy, on the endogenous protective pathway, which limits the in-depth exploration of the value of translational medicine.

## Conclusion

In summary, this study reveals a potential injury–response–protection axis in ischemic stroke: microglia act as core regulators, and the HMBS/POR/TNF molecular module may augment ADO metabolism to initiate endogenous neuroprotection. Experimental validation confirmed that HMBS/POR regulate ADO metabolism by modulating ADA/ADK/SLC29A1/A2 expression, and ADO acts as a functional downstream mediator via A_2_aR signaling. These findings deepen the understanding of IS pathophysiology and provide candidate targets for neuroprotective therapy.

## Data Availability

The original contributions presented in the study are publicly available. This data can be found in the GEO repository with the accession numbers GSE174574 and GSE140275, and the GeneCards database (https://www.genecards.org/ via the search term ‘ADO metabolism’).
